# Social dynamics impact scolding behaviour in captive groups of common ravens (*Corvus corax*)

**DOI:** 10.1186/s12983-022-00477-6

**Published:** 2022-12-12

**Authors:** Christian R. Blum, W. Tecumseh Fitch, Thomas Bugnyar

**Affiliations:** 1 Department of Behavioral and Cognitive Biology, University of Vienna, Vienna, Austria; 2 Haidlhof Research Station, University of Vienna and University of Veterinary Medicine, Vienna, Austria; 3 Vienna CogSciHub, University of Vienna, Vienna, Austria

**Keywords:** Predator recognition, Corvid, Raven (*Corvus corax*), Alarm call, Status signalling, Group dynamics, Learning

## Abstract

**Background:**

Predator avoidance can have immense impacts on fitness, yet individual variation in the expression of anti-predator behaviour remains largely unexplained. Existing research investigating learning of novel predators has focused either on individuals or groups, but not both. Testing in individual settings allows evaluations of learning or personality differences, while testing in group settings makes it impossible to distinguish any such individual differences from social dynamics. In this study, we investigate the effect of social dynamics on individual anti-predator behaviour. We trained 15 captive ravens to recognize and respond to a novel experimental predator and then exposed them to this predator in both group and isolation settings across 1.5 years to tease apart individual differences from social effects and evaluate two hypotheses: (1) weaker anti-predator responses of some individuals in the group occurred, because they failed to recognize the experimental predator as a threat, leading to weak responses when separated, or (2) some individuals had learned the new threat, but their scolding intensity was repressed in the group trials due to social dynamics (such as dominance rank), leading to increased scolding intensity when alone.

**Results:**

We found that dominance significantly influences scolding behaviour in the group trials; top-ranked individuals scold more and earlier than lower ranking ones. However, in the separation trials scolding duration is no longer affected by rank.

**Conclusions:**

We speculate that, while top-ranked individuals use their anti-predator responses to signal status in the group, lower-ranking ravens may be suppressed from, or are less capable of, performing intense anti-predator behaviour while in the group. This suggests that, in addition to its recruitment or predator-deterrent effects, alarm calling may serve as a marker of individual quality to conspecifics.

**Supplementary Information:**

The online version contains supplementary material available at 10.1186/s12983-022-00477-6.

## Background

Successfully recognizing and avoiding predators can have immense fitness consequences [1], but individual variation in anti-predator behaviour remains poorly understood. One well-studied factor is learning to identify predators, which is important to effectively focus anti-predator behaviour on potentially novel threats and to decrease costs of wasted defensive behaviours [2, 3]. Learning can occur at an individual level, providing direct and accurate information, but increasing risk for the observer due to the proximity to the threat. Learning can alternatively occur at a social level, where the sources of information are conspecifics and their responses to the threat. Such social learning reduces the risk to the observer, but also provides potentially less accurate information [4]. Differences in the recognition of and response to predators are further amplified by individual variations in learning accuracy and personality [5, 6]. Considering the evolutionary importance of predation-avoidance, such individual differences may have considerable fitness impacts [2].

A less-studied contributor to individual variation in anti-predator behaviour are social dynamics. Social factors such as sex or dominance might heavily influence individual motivation to participate in anti-predator behaviour [7]. A better understanding of the importance of social dynamics on motivational variation is interesting in its own right, and would also allow better control for motivation when studying variation in learning accuracy. Studies on predator learning to date were either conducted at an individual or a group level (e.g. [8–12]). In the absence of social partners, individual testing may provide similar levels of experienced threat, and therefore similar motivation to engage in anti-predator behaviour, for all subjects. On the other hand, group testing can examine social dynamics and their impact on motivational levels, but they cannot distinguish between whether an individual has failed to learn to recognise a predator, or is simply unmotivated to respond to it. Studies conducted in the wild also face additional difficulties in recognizing individual study subjects (e.g. [13]; but see [14, 15]). Only by combining both group and individual paradigms for the same identifiable individuals can we tease out the specific role of social dynamics on engagement in anti-predator behaviour.

We examined an important anti-predator behaviour—alarm calling—in common ravens (*Corvus corax*), a member of the corvid family. During their early life stages, ravens aggregate in large, mixed-sex, non-breeder groups of varying and inconsistent membership. During the day, they forage in temporary parties of varying sizes and compositions, ranging from as few as two subjects to groups of 20 or even 100 [16–19]. At night some join others to roost in large groups (up to hundreds of individuals). It is during this non-breeder stage that the formation, break-up, and re-formation of bonds and alliances occurs most frequently [19, 20]. Once they reach sexual maturity, at three years, ravens may form long-term pairs, leave other non-breeders, and attempt to occupy a breeding territory of their own, which they defend against other aspiring breeding pairs and groups of non-breeders [21].

When confronted with potential predators, corvids produce harsh alarm vocalisations directed at the predator (“scolding”), presumably both to harass the predator into leaving, and to recruit conspecifics for social support [2]. Such group mobbing can provide learning opportunities for inexperienced individuals [22], and has been shown to indicate alarm callers’ status in several corvid species (white-throated magpie-jays (*Calocitta formosa*) [23]; hooded crows (*Corvus cornix*) [24]; black-billed magpies (*Pica hudsonia*) [25]). While for common ravens this has yet to be shown, we know that individuality is encoded in other raven call types [26–28], and that ravens respond more strongly to alarm calls of adults, than those of juveniles [29].

In a series of elegant studies on wild American crows (*Corvus brachyrhynchos*), Marzluff and colleagues [13, 30] demonstrated that social learning about the potential threat of particular humans occurs, and transmits both horizontally within the local population, and vertically across generations. In those studies, human experimenters could be distinguished via facial masks, and their threat level was manipulated via their initial participation in, or absence from, catching and banding of crows. Using a similar design, we previously demonstrated that members of two captive groups of ravens can remember a ‘dangerous’ human for multiple years [31]. Interestingly, individuals showed considerable variation in their scolding response, and dominance status was a strong predictor for their behaviour. Indeed, dominant individuals (individuals that won the majority of their conflict interactions) took the lead in most scolding bouts, together with their closest affiliates, indicating strong social dynamics effects [31].

But why should dominant ravens differ from subordinates in scolding? A recent study on jackdaws found that the more individuals give an anti-predator response, the more attractive the display becomes to others to join [32] and, presumably, the more likely the predator is to leave. Given that ravens would profit from recruiting conspecifics to participate in anti-predator defence in similar ways, the described dominance-related variation in scolding seems puzzling. One possibility is that, in our previous study [31], not all of the ravens were knowledgeable about the predator stimulus, and that subordinates in particular had not yet learned that the masked human “predator” represents a risk. Another possibility is that social dynamics influence scolding behaviour and although all ravens knew about the predator, some ravens’ responses were suppressed. Some individuals might have been “free-loading” on the anti-predation efforts of others, typically dominants [33].

It is also possible that dominant individuals could afford to show more scolding than subordinates, simply because they were in a better physical condition (see [7]). The ravens’ anti-predator behaviour could thus serve as an honest signal, indicating the callers’ quality (see [34, 35]). Another possibility is that dominants actively suppress calling in subordinates, to highlight or exaggerate their own quality. Preventing others from calling is both energetically costly and takes time away from engaging in the ongoing anti-predator response, thus counteracting the beneficial effects of group mobbing. Hence, such a costly behaviour should occur only in low- to moderate-risk situations, and/or when potential mates are in the audience. Similar status-signalling effects have also been hypothesized for raven recruitment calls at rich but defended food sources [36], where high-status individuals within the non-breeder flock tend to produce more calls.

**Fig. 1:**
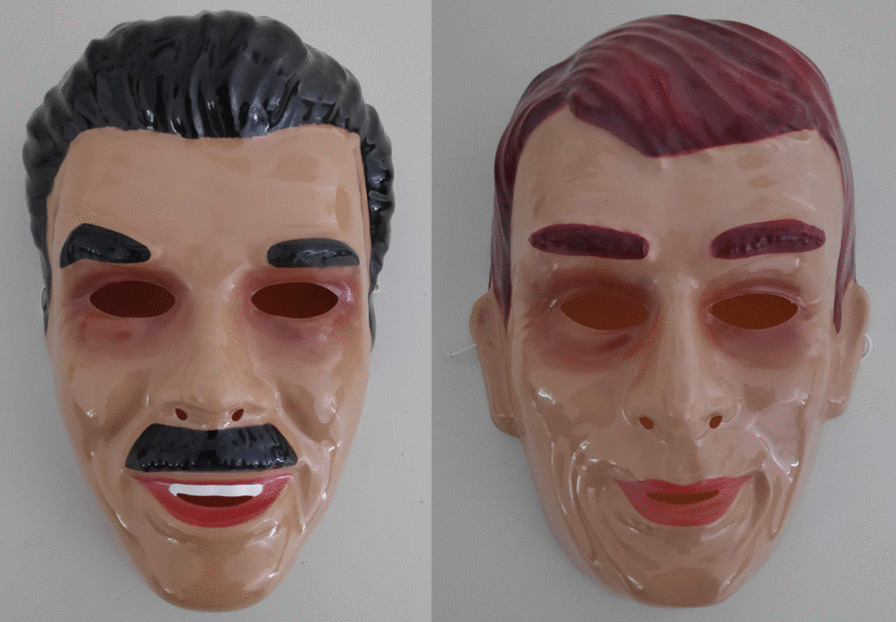
Masks worn during the presentations. The predator mask is on the left, the control mask on the right

In the current study, we experimentally investigated the potential effect of such social dynamics on individual variation in ravens’ scolding behaviour. We followed up on our previous study, in which we trained two groups of eight ravens each to recognize a human wearing a particular mask (Fig. 1) as a potential novel “predator” [31]. During training, the masked person carried a dead raven in their hand, simulating the outcome of a predation event [37]. However, all subsequent test trials were carried out with the masked person only, and without any dead raven. One bird was excluded due to health issues, but the remaining 15 individuals were tested in both group and individual settings. Specifically, we compared scolding responses during six group trials, where motivational levels might be heavily impacted by social dynamics, to the responses in a single separation trial per individual, where any direct social interactions were absent. We based our hypotheses on the considerations mentioned above, specifying effects due to individual learning (or not) and social influences (or their absence). Our two hypotheses are:

- Hypothesis 1: Low scolding durations by some individuals while in the group are not caused by social dynamics, but based on a failure to learn, resulting in some individuals simply not perceiving the artificial predator as a threat.
- Hypothesis 2: Individuals with low scolding durations in the group did learn to recognize the artificial predator as a threat, but their scolding expression is decreased due to social dynamics, specifically their low rank.

Hypothesis 1 predicts the same pattern of calling, in both the group and separation trials, because failure to learn during the group trials would persist into the separation trials, leading to weak or no scolding responses there. Hypothesis 2 predicts different scolding patterns in the separation trial, where previously quiet subjects now would scold with more intensity, because the social dynamics preventing calling in the group condition would be absent in separation.

## Methods

### Subjects and housing

**Fig. 2:**
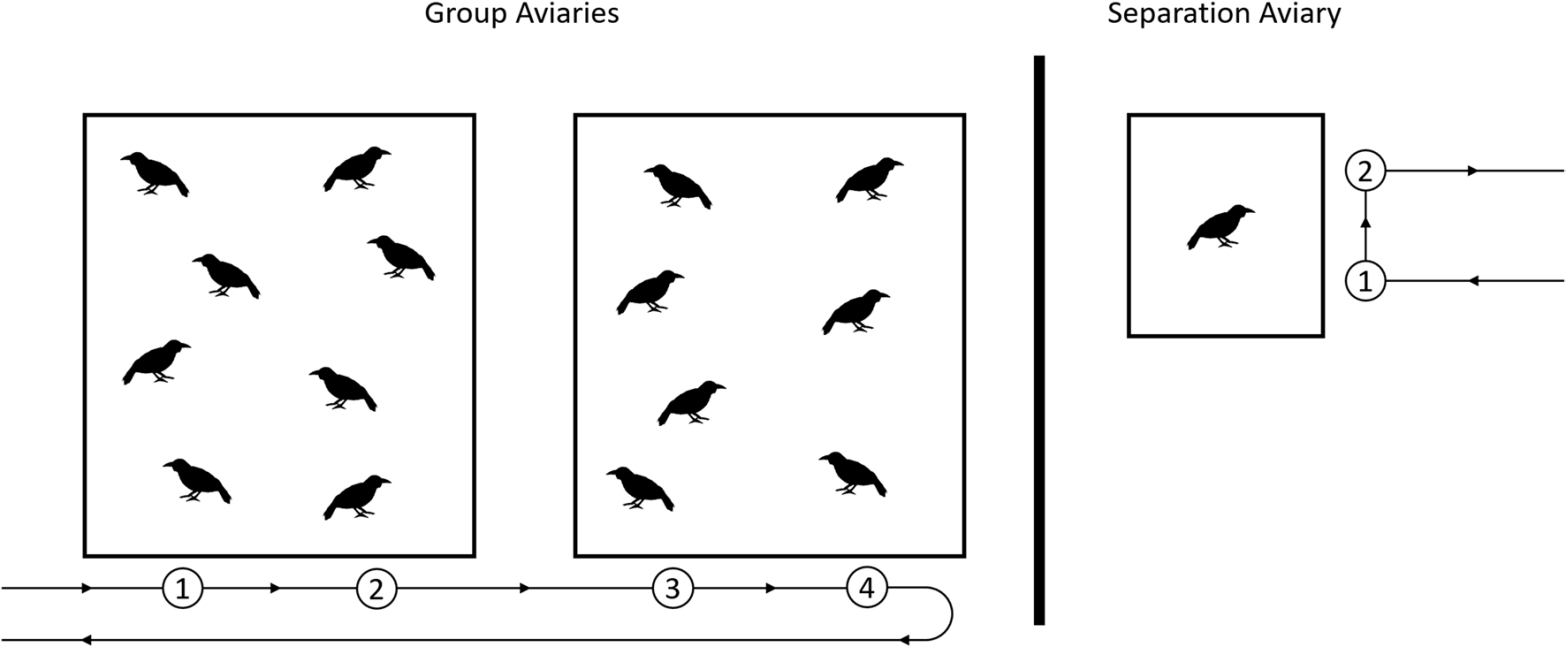
Layout of the aviaries. Group aviaries are depicted on the left, the separation aviary on the right. Presentations were conducted either for both complete groups, or for a single separated raven. The presenter walked along the horizontal line, following the direction indicated by the arrows, and stopped at two locations per aviary (indicated by numbered circles), where they faced the ravens while remaining still for two minutes. The vertical bar represents a visual, but not acoustic barrier between the group and separation aviaries

Study subjects were 15 captive, non-breeding ravens, housed in two groups (Group A: 5 females and 3 males, all parent-raised and hatched in 2010; Group B: 3 females and 4 males, one female hatched in 2010, all others in 2011, 5 hand-raised and 2 parent-raised). The subjects were housed in large, neighbouring outdoor aviaries with walls of wire mesh, netted ceilings and a substrate of wood chips and sand. Branches and plants were provided for perching and enrichment. Smaller, visually isolated compartments made of wood were attached to the aviaries to provide shelter and opportunities for retreat. Food was provided twice a day and consisted of meat, fruits, grain products and vegetables; water was provided ad libitum. All ravens were marked with coloured leg-bands for visual identification. The separation aviary was next to the two main aviaries and allowed audiovocal, but not visual contact (Fig. 2).

### Experimental procedure

The standard procedure for all group trials consisted of a human presenter, wearing standardised clothing (consisting of an olive-grey rain poncho, rubber boots, white gloves and a plastic face-mask), walking first to aviary B, where they remained still for two minutes at two fixed locations on opposite sides of the aviary. They then walked over to aviary A where they repeated the two two-minute presentations and then left the area the same way they came (Fig. 2). Due to the layout of the aviaries, counter-balancing the presentation order was not possible.

During four preparatory training trials in October 2011, the presenter wore a specific “dangerous” mask, and carried a dead raven in their hand. The dead raven was obtained from the Cumberland Wildpark, Grünau, Austria, which is located within a wild raven non-breeder area, and where wild ravens sometimes fall prey to predators or die from other natural causes. Both previous research [37] and our own previous study [31] found pairing a specific human with a dead raven to be highly efficient in eliciting a scolding response from corvids, and encouraged formation of a strong negative association between the potential predation outcome (the dead raven) and the human wearing this particular mask (vs. a control mask).

**Fig. 3:**
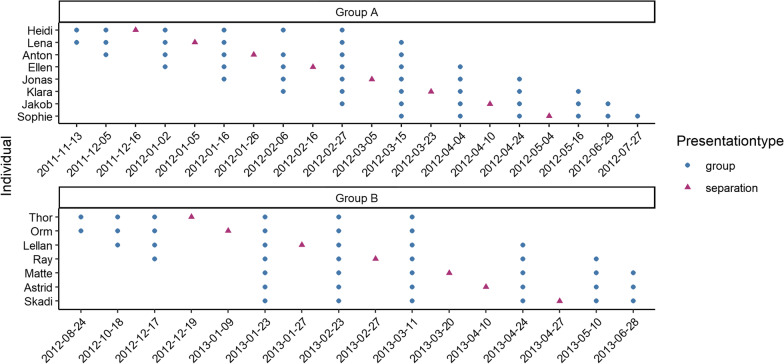
Group and separation trials per subject and date. Every separation trial for every subject was superseded by three group trials and followed by three group trials, with only two exceptions: Heidi had two group trials before and four after her separation trials, because her separation happened just after the training phase (where a dead raven was presented). Skadi had four group trials before and two after, because she left the group soon after her separation. Duration data was extracted per subject for the marked dates. Latency data was extracted for all subjects and all group trials leading up to 2013-02-23, as from that time on subjects of group A were removed from the group to form breeding pairs

After training trials concluded, we began our data collection by continuing these group trials for the next 1.5 years, but now without the dead raven and with no additional training trials. Group trials were conducted approximately every 20 days until May 2012, then every 35 days until June 2013 (Fig. 3). In a previous publication we showed that on a group level, the “dangerous” mask condition elicited significantly longer scolding durations than the control mask condition [31]. In this study, we only use data of group trials conducted with the “dangerous” mask.

In addition to these group trials, we also conducted separation trials, where a single individual was moved from the group to the nearby separation aviary for three days. The separated individual and the remaining group could not see, but could still hear, each other due to the aviary layout. During the separation trials we again presented the “dangerous” mask, but used an unmasked condition as control, to counteract any potential generalisation towards the control mask across 1.5 years. These separation trials were carried out every 20 days from December 2011 to May 2012 for group A, and from December 2012 to April 2013 for group B (Fig. 3). In November 2012 four subjects were removed from group A to form breeding pairs in other aviaries, and the remaining subjects were merged into one large group.

The group trials were presented by different humans, but all separation trials were carried out by the same human (the animal trainer, who also presented in some group trials). Data used for analysis consisted of the single separation trial and the 6 group trials closest to it, spanning on average 138 days per subject (SD = 36 days).

For all trials we video-recorded the birds’ behaviour the entire time the masked presenter was in view of the ravens (Canon Legria HF S10, Canon Legria HF S30), and regularly called out the locations and ID of all group members during filming, to allow individual recognition of all subjects throughout the video. Cameras were operated by researchers that also conducted regular filming of social protocols multiple times a week, and were therefore familiar to the ravens. Camera operators always kept several meters distance to the presenter and stood at the same locations in front of the aviaries that were used for filming social protocols to reduce the chances of the ravens responding to the camera operators. During the entire period of our data collection, ravens never scolded the camera operators, neither during the experiments nor during the social protocols. Afterwards, CRB coded the durations and latencies of the alarm calling behaviour per subject using the software “Solomon Coder” [38] with a precision of 0.2 s. While the ID of the subjects was identifiable throughout the videos, the mask type was tracked separately and not mentioned or visible on the videos, therefore the coder was blind to the test condition, but not to the dominance status of the subjects.

## Statistics

### Scolding duration

The analysis was carried out in R version 3.6.1 [39] using a generalized linear mixed model (function “glmmTMB” from the same-named package; version 1.0.2.1) [40], using a beta-distribution and logit link. As response we included transformed scolding duration as proportion of total duration of predator presentation (which varied slightly across group trials due to different walking speeds of different presenters). If a subject did not scold for the entire trial, it was included in the analysis with a scolding duration of 0. As test predictors we included presentation-type (group vs. separation) and top-ranked (whether an individual was the highest ranking male or female in the dominance hierarchy for its group), plus an interaction between them. Dominance is usually included as ordered hierarchy (e.g., by calculating Elo ratings), but this was not possible here due to lack of data for the specific group compositions and time periods. However, the dominance differences between positions 1 and 2 were much more pronounced than other differences, and could be identified at all times, because other group members almost never initiated antagonistic behaviours against the most dominant individuals, and the most dominant individuals (for the period of our data collection) always won conflicts [41]. We therefore included dominance as a categorical predictor “top-ranked”.

As control predictors we included factors for each subject’s rearing history (hand-raised vs. parent-raised) and sex (female vs. male), as well as a covariate for days since training. A random effect was included for subject ID, with random slopes for presentation-type and days since training. The factor of presentation-type was entered as a dummy variable and the covariate days since training z-transformed to a mean of 0 and a standard deviation of 1 to help model convergence. Sample size consisted of 15 individuals with 6 group trials and 1 separation trial per individual.

For our model diagnostics we confirmed normal distribution of the residuals and of the best linear unbiased predictors by plotting them and visually inspecting them [42, 43]. We tested for collinearity of predictors using variance inflation factors (VIF; using the function “*vif*” of the package “car”; version 3.0.8) on a linear model comprising the same responses and fixed effect predictors [44]. We found that the control predictor “raising” led to slight, potential collinearity issues (max VIF = 2.16), but was still within acceptable limits [45–47]. Model stability was assessed by excluding levels of random effects one at a time and comparing the estimates to those of the full model [48]. This confirmed the model to be stable with the exceptions of the estimates for the effects of Raising and Sex. We therefore dropped Raising from our full model, which also led to better VIFs (max VIF = 1.03). Overdispersion was calculated using a custom function kindly provided by Roger Mundry and showed the model to be under dispersed (dispersion parameter = 0.64), leading to potentially conservative test results.

We compared this full-model to a reduced-model, lacking the interaction, but containing both main effects of the two test predictors and being otherwise identical to the full model, and to a null model comprised of only control predictors using a chi squared test. The full model (χ2 = 25.75, *df* = 2, *p* < 0.001), and the reduced model (χ2 = 24.56, *df* = 1, *p* < 0.001) were significantly better than the null model, but including the interaction did not significantly improve model fit (full vs. reduced model: χ2 = 1.19, *df* = 1, p = 0.28). We then tested the individual fixed effects of both test predictors in the reduced model using likelihood ratio tests [49] by running the drop1 function with the test argument set to “Chisq”. Dominance showed a significant effect (*p* < 0.001) and presentation-type a trend (*p* = 0.076). In the case that the interaction term is not significant, but at least one of its main effects are, a post-hoc test can still be done to understand group differences [50], we therefore continued with the full model.

Finally, we conducted a post-hoc investigation of the different levels of the interaction term by applying a Tukey test with P value adjustment (using the function “emmeans” of the package “emmeans” version 1.6.0) [51] to calculate contrasts in estimated marginal means.

### Scolding latency

We investigated the response of scolding latency in the group trials, using a GLMM with gaussian error distribution and log link, formulated in the package glmmTMB. Individuals that never scolded were not included in the analysis. As test predictor we included an interaction of the factors top-ranked and group, including their main effects. Group A was much further from the starting point of the presenter than group B (and after November 2012 the merged group), therefore an effect on the scolding latency was expected. As control predictors we included fixed effects for sex, group, and days since training. We also added a random effect for subject ID, with random slopes for days since training (z-transformed) and group (dummy coded). This model did not converge, so we removed the interaction term and selected top-ranked as only test predictor and kept group as control predictor. The following model still did not converge, so we removed the random slopes for group, which finally led to convergence.

We again visually confirmed normal distribution of the residuals and the best linear unbiased predictors, tested collinearity using variance inflation factors (max VIF = 2.33, mean VIF = 1.62) and found no problems. We also ran model diagnostics using the package DHARMa (version 0.4.3), which returned non-significant results for the dispersion test (dispersion parameter = 1.01, *p* = 0.85), the KS test (*p* = 0.70) and the outlier test (*p* = 0.48). Model stability was assessed using the same custom function as in the previous model, which revealed the model to be quite unstable, especially for the effects of the predictors group and top-ranked.

We compared this full model to a reduced model, lacking the test predictor top-ranked, and found the full model to be significantly better fitted (χ2 = 9.019, *df* = 1, *p* = 0.003). We therefore kept the full model for the latency analysis.

### Scolding order

**Fig. 4:**
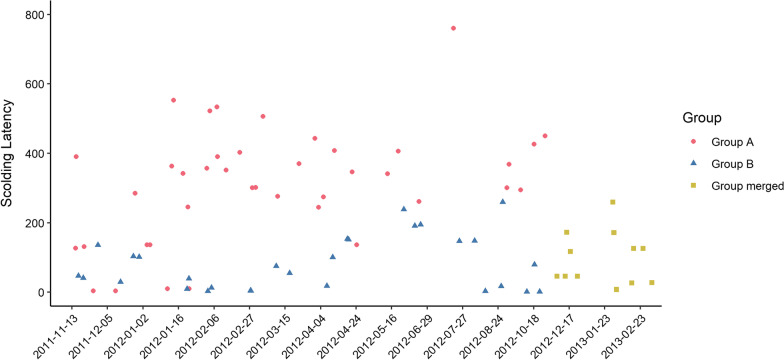
Raw data for scolding latency per group and date. This shows large variation of minimum latencies between trials and mean latencies between groups as potential noise due to the non-counterbalanced path the experimenter walked on

Considering the poor stability of the latency model, we investigated the latency response and found considerable variation across trials (mean = 8.56, SD = 12.16, min = 0.2, max = 45.6), where the minimum latency of some trials was larger than the median latency of other trials (Fig. 4). We believe this noise to be responsible for the low stability and wanted to follow up with another analysis that would avoid this noise. We therefore investigated the scolding order per group, a ranked variable derived from the latency, but which avoided this source of noise, while sacrificing some resolution.

We analysed scolding order using an ordinal logistic regression, using the function “polr” from the package MASS (version 7.3-53.1). As response we included the scolding order per group. As test predictor we included top-ranked, as control predictors we included sex and days since training. We compared this full model to a reduced model lacking the test predictor and found the full model to be significantly better fitted (χ2 = 8.83, *df* = 1, *p* < 0.001). We tested collinearity using VIFs by running a linear model with the same predictors and a dummy response (max VIF = 1.3). We also tested assumption of proportional odds using the function “brant” of the package “brant” (version 0.3-0). Proportional odds assumption held, but fitted probabilities included 0 and 1, so we followed up by manually investigating proportional odds for each scolding order and found that the assumption was no longer met for orders of 4 or higher. Data exploration revealed that 89 out of 98 observations occurred in scolding orders below 4, and that in some instances (e.g. scolding orders of 6 for males) no observations occurred. We therefore removed scolding orders of 4 and above from the test data, ensuring that proportional odds assumption was met throughout, which was also confirmed by rerunning the function “brant”. This reduced our number of subjects from 12 to 11. In addition to the standard output for the full model, we also calculated odds ratios and confidence intervals by exponentiating the estimates and confidence intervals.

## Results

### Scolding duration

**Fig. 5:**
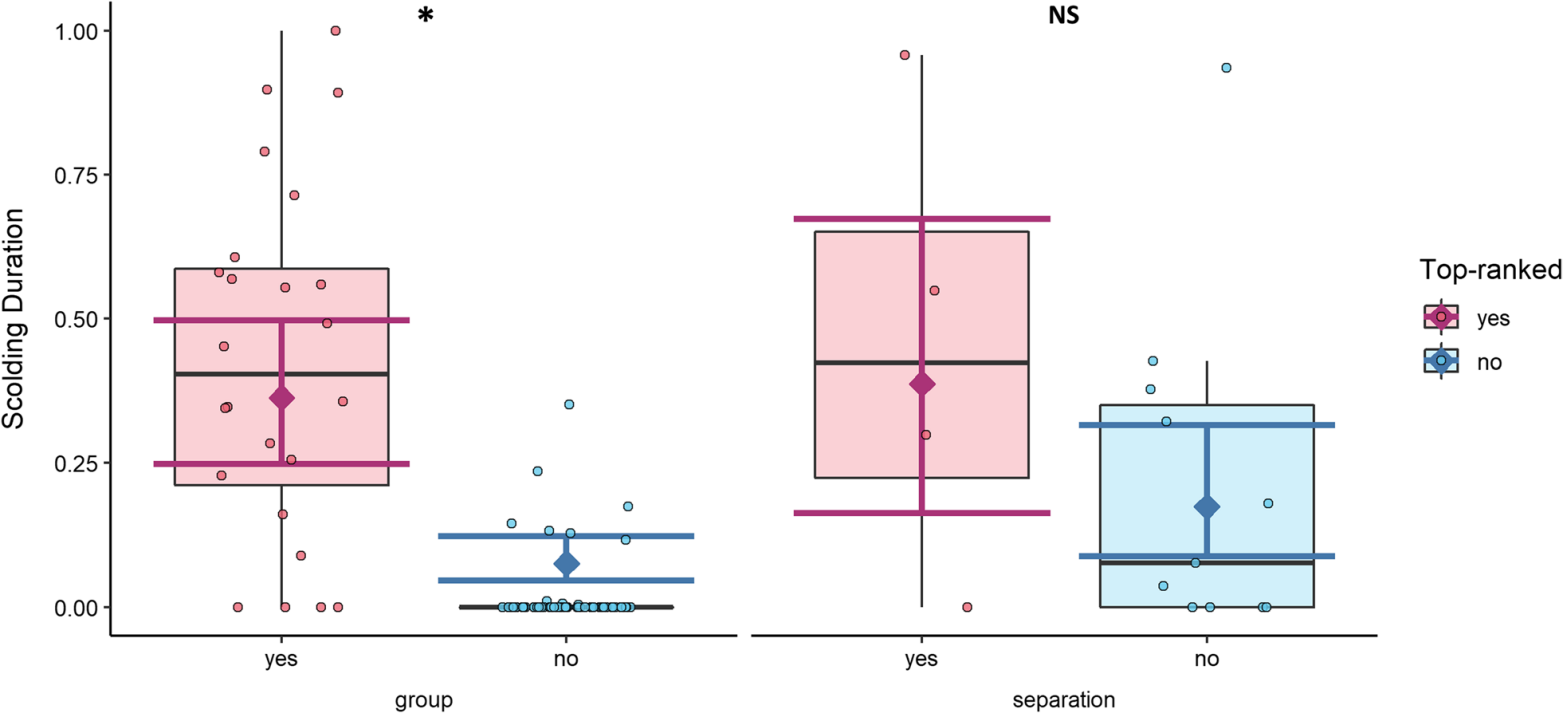
Scolding duration per presentation-type (group vs. separation) and top-ranked (yes vs. no). Raw data is depicted as boxplot and scatterplot, model estimates and 95% confidence intervals as diamonds and error bars. Top ranked individuals scolded significantly longer in the group trials, but not in the separation trials

The model identified both main effects and the control predictor “days since training” as significant, but not the control predictor “sex” (Table 1, Fig. 5). Post-hoc testing showed that top ranked individuals scolded longer than others in the group (post-hoc: E = − 1.94, SE = 0.32, *p* < 0.001) but not in separation (post-hoc: E = − 1.10, SE = 0.70, *p* = 0.40).

**Table 1:** Model results for scolding duration

Explanatory variables	Estimate (95% CI)	SE	z value	*p* value	
(Intercept)	− 2.50 (− 3.04; − 1.96)	0.27	− 9.1	< 0.001	***
Presentation-type separation	0.94 (0.10; 1.78)	0.43	2.2	0.03	*
Top-ranked yes	1.94 (1.31; 2.57)	0.32	6.04	< 0.001	***
Sex male	0.11 (− 0.36; 0.58)	0.24	0.47	0.64	
Days since training	− 0.29 (− 0.55; − 0.03)	0.13	− 2.19	0.03	*
Presentation-type separation: Top-ranked yes	− 0.84 (− 2.33; 0.65)	0.76	− 1.1	0.27	

Significance codes:. < 0.1; *< 0.05; **< 0.01; ***< 0.001", where "." corresponds to values smaller than 0.1; "*" to values smaller than 0.05

Reference categories are “group” for “Presentation-type”, “no” for “Top-ranked”, and “female” for “Sex”. N(observations) = 105, N(subjects) = 15. Significance codes:. < 0.1; *< 0.05; **< 0.01; ***< 0.001. Post-hoc testing on the interaction showed top ranked individuals scolded longer in the group (E = − 1.94, SE = 0.32, *p* < 0.001) but not in separation (E = − 1.10, SE = 0.70, *p* = 0.40)

### Scolding latency

**Fig. 6:**
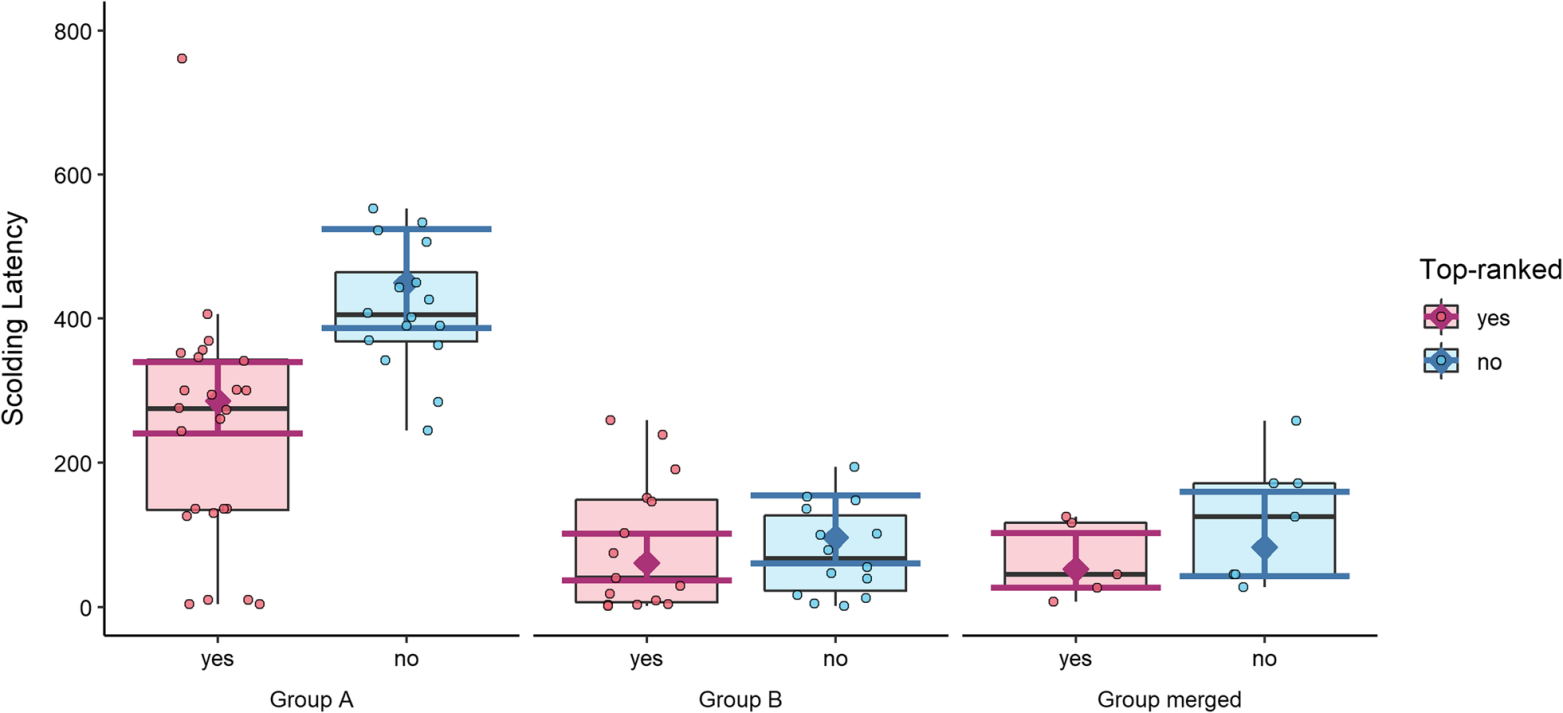
Scolding latency per group (A, B, merged) and top ranked (yes, no). Raw data is depicted as boxplot and scatterplot, model estimates and 95% confidence intervals as diamonds and error bars. Top ranked individuals scolded significantly earlier across all group trials

Top-ranked subjects had a significantly lower latency to scold. We also found that group A scolded significantly later than both group B, and the merged group. Again, “days since training” was significant, but “sex” was not (Table 2, Fig. 6).

**Table 2:** Model results for scolding latency

Explanatory variables	Estimate (95% CI)	SE	z value	*p* value	
(Intercept)	5.85 (5.63; 6.07)	0.11	52.74	< 0.001	***
Top-ranked yes	− 0.45 (− 0.66; − 0.25)	0.11	− 4.30	< 0.001	***
Group B	− 1.54 (− 2.03; − 1.05)	0.25	− 6.19	< 0.001	***
Group merged	− 1.69 (− 2.36; − 1.03)	0.34	− 5.00	< 0.001	***
Sex male	− 0.06 (− 0.3; 0.17)	0.12	− 0.53	0.59	
Days since training	0.00 (0.00; 0.00)	0.00	2.53	0.01	*

Significance codes:. < 0.1; *< 0.05; **< 0.01; ***< 0.001", where "." corresponds to values smaller than 0.1; "*" to values smaller than 0.05

Reference categories are “no” for “Top-ranked”, "A" for "Group", and “female” for “Sex”. N(observations) = 81, N(subjects) = 12. Significance codes:. < 0.1; *< 0.05; **< 0.01; ***< 0.001

### Scolding order

**Fig. 7:**
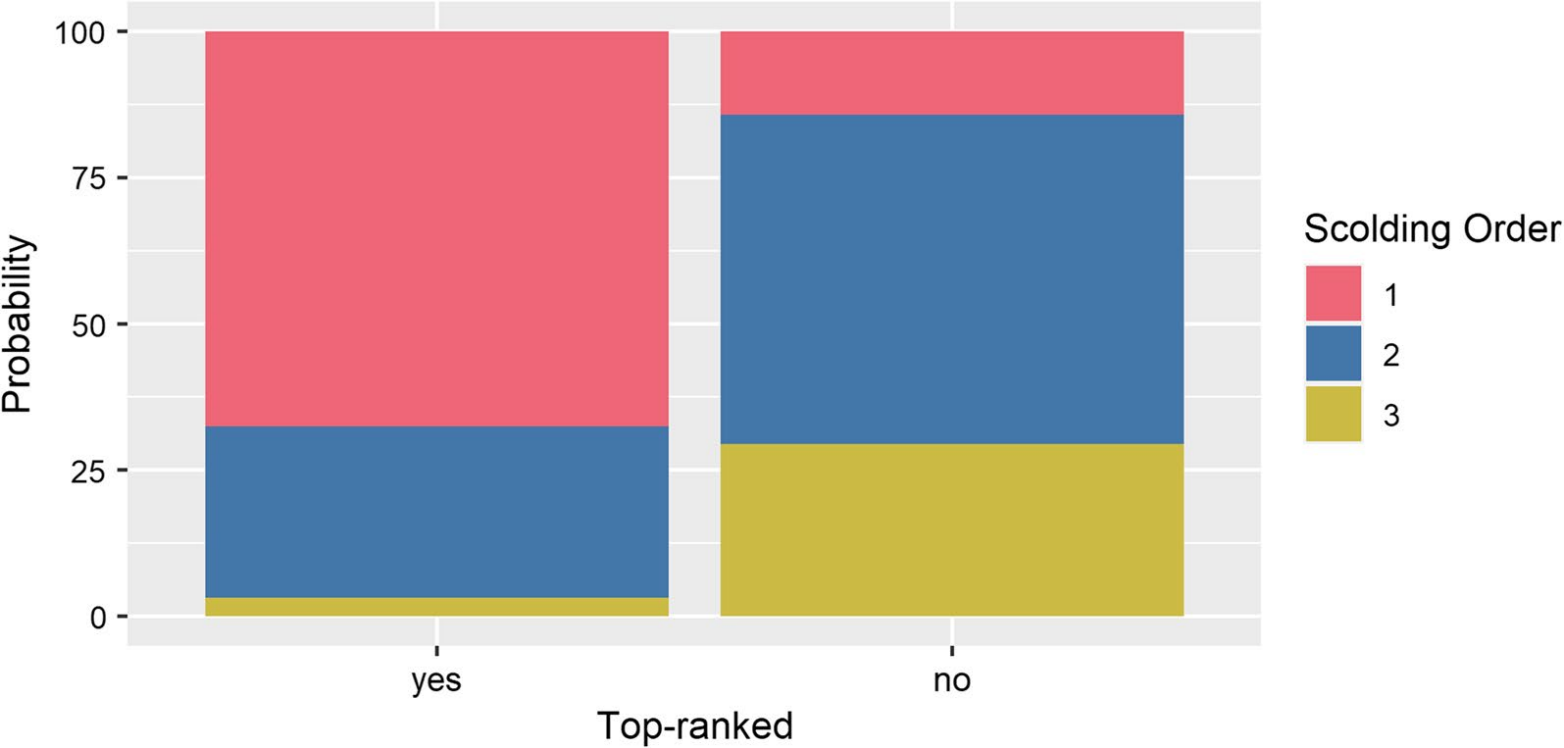
Probabilities for scolding orders 1, 2 and 3 for top ranked and lower ranked subjects. Top ranked individuals scolded significantly earlier across all group trials. Scolding orders higher than 3 were excluded because they did not meet model assumptions

Top-ranked subjects had a significantly lower scolding order, and we found no significant effects of our control predictors sex and days since training (Table 3, Fig. 7).

**Table 3:** Model results for scolding order

Explanatory variables	OR (95% CI)	Value	SE	t value	*p* value	
Top-ranked yes	0.08 (0.02; 0.26)	− 2.54	0.64	− 3.96	< 0.001	***
Sex male	0.77 (0.26; 2.31)	− 0.26	0.56	− 0.47	0.64	
Days since training	1.00 (1.00; 1.00)	0.00	0.00	− 0.51	0.61	

Significance codes:. < 0.1; *< 0.05; **< 0.01; ***< 0.001", where "." corresponds to values smaller than 0.1; "*" to values smaller than 0.05

Reference categories are “no” for “Top-ranked” and “female” for “Sex”. N(observations) = 72, N(subjects) = 11. Significance codes:. < 0.1; *< 0.05; **< 0.01; ***< 0.001

## Discussion

To our knowledge, this is the first study that compared anti-predator reactions in both group and separation settings of individuals that had learned to recognize a novel predator in a group setting. While top-ranked subjects scolded significantly longer in the group, this was no longer the case in separation. The fact that most subordinates (i.e., all but the top-ranked individuals) called in the separation trials indicates that they had indeed learned to recognize the artificial predator. These findings thus speak against hypothesis 1 (failure to learn about the predator), but are consistent with hypothesis 2 (social dynamics affect scolding behaviour). Furthermore, the control trials (with unmasked presenter) did not elicit a single alarm call throughout all separation trials, indicating that these lower-ranked birds indeed learned about and responded to the presence of the “dangerous” mask, rather than the unfamiliar solitary setting, the absence of conspecifics, or other extraneous factors. We can therefore also exclude any effects of idiosyncrasies of the presenter, such as stature, gait or walking speed.

It is conceivable that separation could contribute to greater reaction from the individuals, not because they are repressed when in the group, but because they are simply more stressed and aroused when they have to face a threat alone. While plausible, there are several reasons to doubt this. First, if this hypothesis were correct, we would expect isolation to have the same effect on all subjects, but we found the effect only for the lower-ranked (non-dominant) individuals. Second, not all birds necessarily experienced separation as stressful: a parallel study focused on hormonal and behavioural indicators of stress found that only those birds that were socially well-integrated showed elevated stress levels during separation, whereas the reverse pattern was true for socially less-integrated birds [52]. One might still argue that even individuals that were less stressed in isolation as compared to in the group might find facing a threat alone more stressful, and therefore engaged in increased calling. Future studies need to address this possibility, e.g. by gathering independent measures of physiological stress levels in both situations.

The social dynamics hypothesis is quite general, and our results are in principle compatible with multiple more detailed explanations. Subordinates may free-load on already scolding top-ranked, dominant subjects. Alternatively, dominants may use the anti-predator context for showing off, and subordinates may be unable to afford this activity, or are actively supressed by dominants from doing so. Disentangling these potential underlying causes is difficult and will require further research. However, we find little support for the “free-loading” explanation, and tentative support for the two latter possibilities. Note that our experimental set-up allowed the individually separated ravens to remain in auditory contact with their group members. During individual tests with the masked human experimenter, group members could thus join the separated birds in scolding, which they regularly did. This observation speaks against the free-loading argument, as in the separation trials scolding conspecifics were also close by. However, the distance to the nearest scolding conspecific was larger in separation trials than in group trials and the visibility to the conspecifics was obstructed, possibly favouring free-loading in one condition more than in the other.

Turning to the show-off interpretation, we found that dominant individuals also had lower scolding latencies and scolding orders in a group setting, while subordinate birds scolded later, if at all. This pattern is in line with the possibility that dominants used scolding to showcase their individual quality, not only by scolding longer but also by scolding earlier than individuals of lower rank. What does not entirely fit this interpretation is that some subordinate individuals, like the female Skadi in Group B, do repeatedly engage in scolding. Raising could be a possible explanation for this, as e.g. Skadi was one of two parent-raised subjects in her group of mostly hand-raised birds, and it has been shown that hand-raising has an impact on raven social behaviour in later life [53]. Additionally, it seems reasonable to assume that subjects raised by humans perceive humans in general as lower threat, which might impact our findings. It was not possible to include raising type as predictor in this study due to collinearity and model stability issues, but this possibility certainly merits further investigation.

Finally, we occasionally observed dominant individuals attacking subordinates when those engaged in intense scolding, hinting towards active suppression of subordinate’s anti-predator behaviour. Although it seems difficult to explain why dominants should do so, our experimental paradigm might have favoured status-signalling, as it provided a context where predators posed a low risk and potential mates were present in the groups. Generally, this show-off interpretation is in line with previous publications on corvids suggesting that alarm calling is linked to dominance [7, 34], social rank and recent mating success ([54, 55]; but see [56]). They also resemble findings in cowbirds, in which dominant males have been observed to prevent subordinates from singing and courting females [57–60]. Furthermore, when dominant male cowbirds were removed from the group, subordinates increased their singing rates [60]. This mirrors the pattern we observed in our separation trials, and further supports the suggestion that raven scolding may function (among other purposes) as status signalling.

Given that our attempts to differentiate between the free-loading and the two types of status-signalling hypotheses are based on fragile evidence, follow-up studies will be required to clearly disentangle those causes. For instance, future studies could investigate individuals’ scolding responses in the group when the top ranked male and female are removed, or use sound-isolated separation aviaries. Being kept in captivity and with regular contact to humans, a human-shaped “predator” might be highly familiar and thus pose a rather low threat level and elicit a weaker alarm response by captive groups [61–63]. Repeating these experiments with a different predator stimulus of higher perceived threat level, or in the wild, and comparing the results might shed additional light on social aspects of scolding behaviour.

Taken together, our results indicate that dominance-dependent differences in scolding duration observed in group alarm-calling vanish during individual separation. We therefore conclude that low scolding in the group setting by subordinates is unlikely to be caused by lack of learning. Until now, scolding behaviour was mainly understood to serve, (1) predator deterrence, e.g. to harass and deter the predator by alerting it of its detection [1, 2], (2) social learning opportunities, e.g. transmitting information about predators to inexperienced conspecifics [30, 37], and (3) recruiting social support [64–66]. These three established functions of scolding fail to explain our pattern of results. Exposure to and risk from the artificial predator increased to the same degree for all ravens when separated, yet only subordinates increased their response. We therefore suggest that social dynamics within the group influence individual scolding behaviour, specifically that of subordinates, possibly because dominant individuals employ their intense scolding displays as a signal of high social status, and suppress calling by subordinates. Alternatively, low-status individuals cannot afford intense scolding due to energetic constraints, and freeload on those that do. Either way, the current data suggest that, in addition to its direct deterrence effects on the predator, or its recruitment effects on conspecifics, alarm calling in social contexts might play an important signalling role indicating individual quality.

## Conclusions

We compared the scolding behaviour of 15 common ravens (*Corvus corax*) towards a learned threat in both group and isolation settings. We found that scolding varied strongly between individuals in the group setting, where top ranked individuals scolded significantly longer. However, when separated, this rank effect was no longer observed. We argue that the low scolding participation of some individuals in the group setting was caused, not by a lack of learning about the potential threat, but by social dynamics which were absent in the separation trials. This raises the possibility that scolding behaviour may serve as a marker for individual status, in addition to its well-established functions of recruitment, predator deterrence, and facilitating social learning.

## Supplementary Information

**Additional file 1: Table S1**. Data used for the analysis on scolding duration.

**Additional file 2: Table S2**. Data used for the analysis on scolding latency and order.

**Additional file 3**. R script used for statistical analysis and plotting.

## Data Availability

All data generated or analysed during this study and the R script are included in this published article [and its Additional files 1, 2, 3].
